# Population Structure, Diversity and Reproductive Mode of the Grape Phylloxera (*Daktulosphaira vitifoliae*) across Its Native Range

**DOI:** 10.1371/journal.pone.0170678

**Published:** 2017-01-26

**Authors:** Karl T. Lund, Summaira Riaz, M. Andrew Walker

**Affiliations:** Department of Viticulture and Enology, University of California, Davis, California, United States of America; National Cheng Kung University, TAIWAN

## Abstract

Grape Phylloxera, *Daktulosphaira vitifoliae*, is a gall-forming insect that feeds on the leaves and roots of many *Vitis* species. The roots of the cultivated *V*. *vinifera* cultivars and hybrids are highly susceptible to grape phylloxera feeding damage. The native range of this insect covers most of North America, and it is particularly abundant in the eastern and central United States. Phylloxera was introduced from North America to almost all grape-growing regions across five of the temperate zone continents. It devastated vineyards in each of these regions causing large-scale disruptions to grape growers, wine makers and national economies. In order to understand the population diversity of grape phylloxera in its native range, more than 500 samples from 19 States and 34 samples from the introduced range (northern California, Europe and South America) were genotyped with 32 simple sequence repeat markers. STRUCTURE, a model based clustering method identified five populations within these samples. The five populations were confirmed by a neighbor-joining tree and principal coordinate analysis (PCoA). These populations were distinguished by their *Vitis* species hosts and their geographic locations. Samples collected from California, Europe and South America traced back to phylloxera sampled in the northeastern United States on *V*. *riparia*, with some influence from phylloxera collected along the Atlantic Coast and Central Plains on *V*. *vulpina*. Reproductive statistics conclusively confirmed that sexual reproduction is common in the native range and is combined with cyclical parthenogenesis. Native grape phylloxera populations were identified to be under Hardy-Weinberg equilibrium. The identification of admixed samples between many of these populations indicates that shared environments facilitate sexual reproduction between different host associated populations to create new genotypes of phylloxera. This study also found that assortative mating might occur across the sympatric range of the *V*. *vulpina* west and *V*. *cinerea* populations.

## Introduction

Grape phylloxera, *Daktulosphaira vitifoliae* (Fitch) is an aphid-like root and leaf-feeding insect that is now found in most of the world’s vineyards. Phylloxera gained the reputation as the most important viticultural insect pest after they were accidentally introduced from their native home in North America into France during the mid 1800s [1, 2]. There they found a highly susceptible host, the European grape, *Vitis vinifera*. The leaves of *V*. *vinifera* grapes are relatively tolerant to phylloxera feeding, but their fine and structural roots are highly susceptible [1]. Feeding on the young root tips causes hooked galls (nodosities) and feeding on mature structural roots causes large swollen galls (tuberosities), which eventually deform and crack. Both forms of root damage provide entry to soil-borne fungi and bacteria, resulting in progressively more severe root damage, yield loss and eventual vine death [2]. The introduction of grape phylloxera into Europe resulted in the rapid destruction of vineyards and the collapse of a wine based agricultural economy to such a great extent that it was referred to as the “great French wine blight.” Within 30 years more than 90% of French vineyards were affected by the pest [3]. From France, phylloxera spread across Europe and eventually to grape growing regions around the world. Years of research determined that grafting the susceptible *V*. *vinifera* cultivars onto rootstocks derived from resistant North American *Vitis* species, which allow feeding on young root tips and leaves, but prevent destructive feeding on the structural roots, was the only effective means of control.

The native range of grape phylloxera extends from southern Canada to northern South America, but they are most common in the eastern and central United States from Texas to Canada and east to the Atlantic Ocean. Grape phylloxera can also be found in geographically isolated locations in the southwestern United States. The classic life cycle of phylloxera is comprised of cyclic parthenogenesis with temporal polyphenism [2, 4]. The mode of phylloxera reproduction in its native range is postulated to be sexual, based on the observations of different sexual forms of phylloxera in an earlier study; however the efficacy of meiotically produced eggs is in question [2, 5]. It is also not known whether changes to the climate or alterations in host species have any influence on grape phylloxera’s reproductive mode in its native range. In its introduced range, reproduction is thought to be predominantly asexual as reported by researchers in Australia [6, 7, 8], Europe [9], and California [10]. Temperature was found to influence the survivability of crawlers (nymphs) and asexual eggs [11].

Across its native range, phylloxera feed on young leaves and root tips of American grape species [4, 12, 13]. The large number of genetically diverse American grape species and the highly variable environments they occupy plays an important role in the genetic diversity of phylloxera. Like many other herbivorous insects that have evolved specific host-based races [14, 15, 16], grape phylloxera have also evolved distinct host associated races. There have been relatively few studies of phylloxera’s genetic diversity across its natural range. Lin et al. [17] examined phylloxera’s genetic diversity using RAPD markers. They compared phylloxera from three sites in Arizona on the host species *V*. *arizonica* and six sites in New York on the host species *V*. *riparia*. The samples collected from Arizona were strongly associated with collection site and specific host plant. Later, Downie et al. [12] used RAPD markers to examine 98 samples collected across phylloxera’s native range, including samples from the introduced range to determine their point of origin. The RAPD marker data found a strong association by host and secondary groupings by geography. Mitochondrial sequencing also detected host and geographic groupings, but to a lesser extent [13].

DNA marker based studies from samples collected in the introduced range also show evidence of the selective pressure of different host plants [1, 7, 8, 9, 18]. Host based selection of grape phylloxera has been detected, most notably in association with the rootstock AxR#1. This rootstock was originally considered to have adequate resistance to phylloxera [19], but failed in the 1980s after about 15 years of large-scale commercial use in California vineyards. Subsequent research found that two different feeding types existed in California: those incapable of feeding on AxR#1 (biotype A), and those capable of feeding on the structural roots of AxR#1 (biotype B) [20]. More recently, genetically diverse phylloxera strains in Australia were found to differ in their ability to reproduce on *V*. *vinifera* [21], indicating the importance of both genetics and host adaptation.

Grape phylloxera is an appealing insect for the study of the evolution and maintenance of sex because of its ability to have both sexual and asexual life cycles [2]. They are also excellent candidates to study the model of sympatric divergence and speciation because of their intimate association with host plants and their capacity to develop specialized host races. A better understanding of grape phylloxera’s genetic diversity, its genetic structure, factors that influence reproduction (asexual and sexual), patterns of dispersal and gene flow over time in its natural habitat is necessary to develop effective pest management strategies. In this study, more than 500 phylloxera samples were collected from multiple *Vitis* species hosts across the grape phylloxera’s native range. Samples from the introduced range in the United States (California) and from other countries in Europe and South America were also included. The objectives of the study were to assess the genetic diversity of grape phylloxera in its native range and to define boundaries of host and geographic associations; to evaluate grape phylloxera’s mode of reproduction in relation to variable host plants, climates and geographic regions; and finally to establish and evaluate the point of origin of grape phylloxera currently present in California, Europe and South America.

## Results

### Identification of unique MLGs

A set 549 samples collected from phylloxera’s native range in the USA, and samples from California, South America and Europe were genotyped with 32 SSR markers. Six markers were eliminated for reasons explained in the Methods section. Three alleles were observed in 134 samples with one or more markers resulting in 176 triploid data points (S1 Table). More than two alleles can be observed if the genomic site of a SSR primer is duplicated. When this occurred the triploid data points were rechecked to assure accurate scoring. The third allele for the triploid data points was not a unique private allele in the study set except for two alleles at two markers that were not observed in rest of the study set. No correlation was observed between the occurrence of triploid data and the host *Vitis* species. However, samples collected from Arizona, New Mexico, South Dakota and Utah had more triploid data points compared to samples from other regions. When more than two alleles were observed, data was considered missing for that genotype at that marker. In next stage, a total of 47 samples were removed from the study that had missing data for more than three markers. From the remaining 502 samples, a total of **466** unique MLGs were identified with data from 26 SSR markers (S2 Table). Four hundred thirty eight samples were identified as unique MLGs; 58 other samples accounted for **25** MLGs where multiple sampling was carried out on the same plant (the results on the effects of clonal MLGs are presented in the reproductive mode section below); four samples that were collected from different sites and separated by large distances accounted for **2** unique MLGs (California and Peru samples matched including a triploid allele at the Phy_III_19 marker and samples collected from Indiana and Texas matched); lastly two samples collected from South Dakota from the same site but different plants constituted **1** MLG (Table 1). We ruled out the possibility of sample contaminations as samples were processed in different groups for phylloxera extractions and genotyping, thus making cross contamination very unlikely. The Indiana sample that matched the sample from Texas was collected at a vineyard and the chance of contamination due to human movement is possible. The California and Peru samples matched at 53 alleles. The P_sex_ values for all clonal MLGs indicate that they were true clonal samples (the result of asexual reproduction) and not the result of independent sexual events (data not shown).

**Table 1:** The 28 clonal multi-locus genotypes (MLG) found in the full data set. The first two letters of each Sample ID indicates the state the sample was collected in and the host is noted in column 3.

MLG ID	Matching on Same Plant	Host plant
MLG-018	AR0711143, AR0711144	*V*. *vulpina*
MLG-127	MO0751213, MO0751214	*V*. *aestivalis*
MLG-155	MO0801322, MO0801324	*V*. *vulpina*
MLG-167	NC0510783, NC0510785	*V*. *vulpina*
MLG-171	NC0510793, NC0510794	*V*. *vulpina*
MLG-174	NC0520802, NC0520803, NC0520806	*V*. *vulpina*
MLG-176	NC0530811, NC0530812	*V*. *vulpina*
MLG-239	NY0200251, NY0200253	*V*. *riparia*
MLG-244	NY0220272, NY0220273	*V*. *riparia*
MLG-248	NY0240291, NY0240293	*V*. *riparia*
MLG-260	OK0811334, OK0811335	*V*. *vulpina*
MLG-263	OK0821343, OK0821344, OK0821345	*V*. *vulpina*
MLG-267	PA0270323, PA0270324	*V*. *riparia*
MLG-301	TN0590922, TN0590924	*V*. *vulpina*
MLG-310	TN0600942, TN0600943	*V*. *labrusca*
MLG-325	TN0630982, TN0630984	*V*. *vulpina* × *V*. *riparia* hybrid
MLG-331	TN0641001, TN0641003, TN0641004	*V*. *vulpina* × *V*. *riparia* hybrid
MLG-345	TN0661042, TN0661043	*V*. *vulpina*
MLG-349	TN0661052, TN0661053, TN0661054, TN0661056, TN0661057	*V*. *vulpina*
MLG-366	TX0851383, TX0851384	*V*. *vulpina*
MLG-382	VA0420593, VA0420594	*V*. *vulpina*
MLG-383	VA0430601, VA0430602, VA0430603	*V*. *vulpina*
MLG-407	VA0480712, VA0480714, VA0480715	*V*. *vulpina*
MLG-409	VA0490721, VA0490722	*V*. *vulpina*
MLG-426	WV0400533, WV0400534	*V*. *vulpina*
Matching at Same Site		
MLG-279	SD0370473, SD0370493	American Hybrids
Long distance matches		
MLG-072	IN0290372, TX0851394	Foch & *V*. *vulpina*
MLG-435	CA0020021, PER1101851	Chardonnay & Torrontel

### Population structure analysis

The first STRUCTURE output indicated a total of 4 populations within 466 MLGs supported by both delta K and Ln P(D) plateau. A closer examination of the individual replicated STRUCTURE runs indicated that population assignment of unique MLGs in two populations was stable and did not change within different replicated runs. However, the position of 34 samples switched between two other populations during the replicated STRUCTURE runs. A second STRUCTURE analysis with 175 MLGs from the two populations in question divided the samples into three distinct populations. The 34 samples that switched groups in the first STRUCTURE analysis formed a new population. We combined the STRUCTURE results from the two analyses and designated five populations within the 466 unique MLGs. Four hundred twenty-five samples (91.2%) were assigned to one of the 5 populations with a STRUCTURE q value of 0.90 or above. An additional 17 samples (3.6%) were associated with one of the 5 populations, but at a lower STRUCTURE-based probability (q was between 0.70 and 0.89). Only 24 samples (5.1%) were admixture samples with no population assignment (a STRUCTURE q value of less than 0.7 for any of the five populations) including four samples that did not group consistently across the analyses. Two of the admixed samples grouped with the *V*. *vulpina* west population, and the other grouped in association with the *V*. *riparia* population.

**Fig 1:**
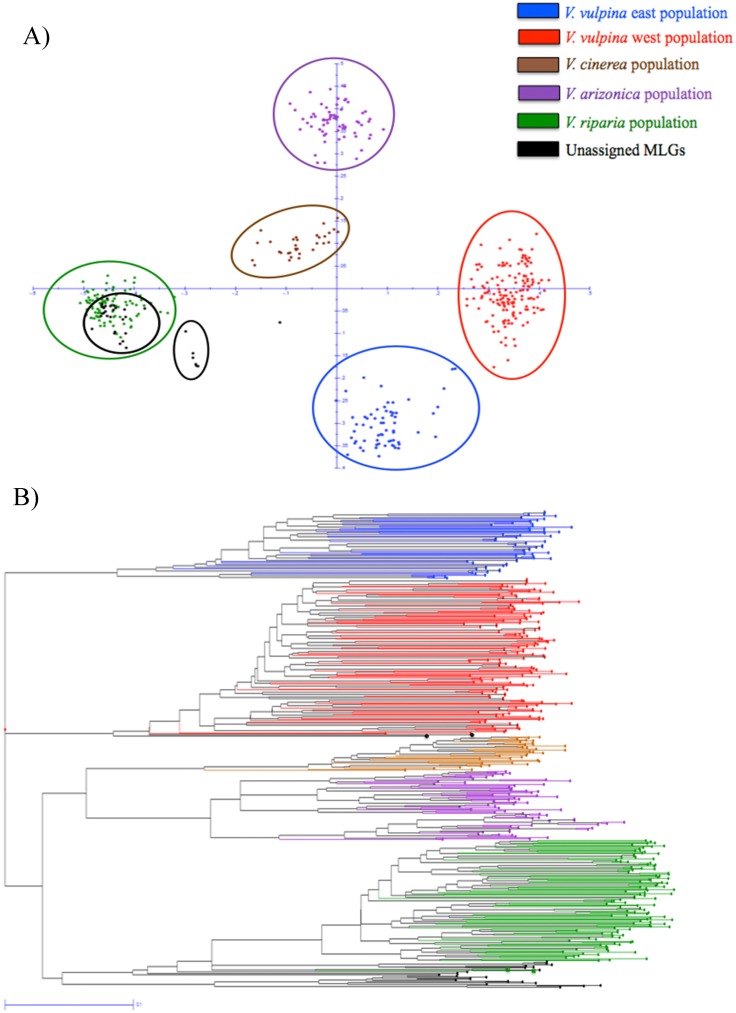
**A) Principal Coordinate Analysis (PCoA) of grape phylloxera MLGs that were assigned to five populations based on the q > 0.9 or that two STRUCTURE analyses (q > 0.7) found were associated**. The X-axis accounts for 22.51% of the variation, while the Y-axis accounts for 8.86%. Samples from the introduced range (Austria, Brazil, Hungary, Uruguay and the California SAL (single adult lineage) lines 101R1, 101R2, FreR1 and FreR2B) grouped within the *V*. *riparia* population marked with the green circle. Samples within the smaller black circle (closer to *V*. *riparia* population) are from Argentina and Peru, and include the California SAL lines AxRR1 and VinR1, which grouped as an admixture between the *V*. *riparia* and *V*. *vulpina* east populations. The California foliar sample WEO4802 (un-circled black dot) was found to be an admixture between the *V*. *riparia* and *V*. *vulpina* west populations. B) Neighbor-joining tree constructed from 466 unique MLGs. Samples that were determined to be an admixture of two populations by STRUCTURE analysis with q-values less than 0.7 were not assigned to any population and are presented in black. Two samples in black circles within the *V*. *vulpina* west population were not considered part of that group by STRUCTURE analysis, however both neighbor-joining tree and PCoA considered them part of *V vulpina* west population. Samples in green circles within the unassigned MLGs were considered to be part of the *V*. *riparia* population by STRUCTURE, and neighbor-joining tree and PCoA placed them within unassigned admixed samples.

The five clusters obtained from the STRUCTURE analysis were also confirmed with the PCoA and neighbor joining tree analysis (Fig 1A & 1B). The PCoA analysis was carried out with MLGs that were conclusively part of a population (Q > 0.90) or were associated with that population (Q > 0.70) (Fig 1A), and with all 466 unique MLGs (S1 Fig). Results from both PCoAs corroborated 5 distinct populations and verified placement of the admix samples. The results were comparable with the neighbor joining tree analysis (Fig 1B). Two of the admix samples again grouped with the *V*. *vulpina* west population, and other two were associated with the *V*. *riparia* population group. Samples from Hungary, Austria, Brazil and Uruguay were all members of the *V*. *riparia* population. California SAL samples that were taken from the rootstocks 101-14Mgt (101-R1 and 101-R2) and Freedom (Fre-R1 and Fre-R2B) were also members of the *V*. *riparia* population. Samples from Peru, Argentina and two other California SAL lines (Vin-R1 and AxR-R1) clustered together as a small group near the *V*. *riparia* population in the PCoA (Fig 1A). The STRUCTURE results suggested that these samples were admixtures between the *V*. *riparia* and the *V*. *vulpina* east populations (q values 0.3 to 0.6). The California foliar sample (WEO4802) was identified by STRUCTURE as an admixture between the *V*. *riparia* and the *V*. *vulpina* west populations (q values 0.4 to 0.5) and was not associated with other samples in the PCoA. The samples identified as admixtures by STRUCTURE were found outside the 5 distinct populations when the PCoA was carried out on all unique MLGs (S1 Fig).

### Host associated population distribution

**Fig 2:**
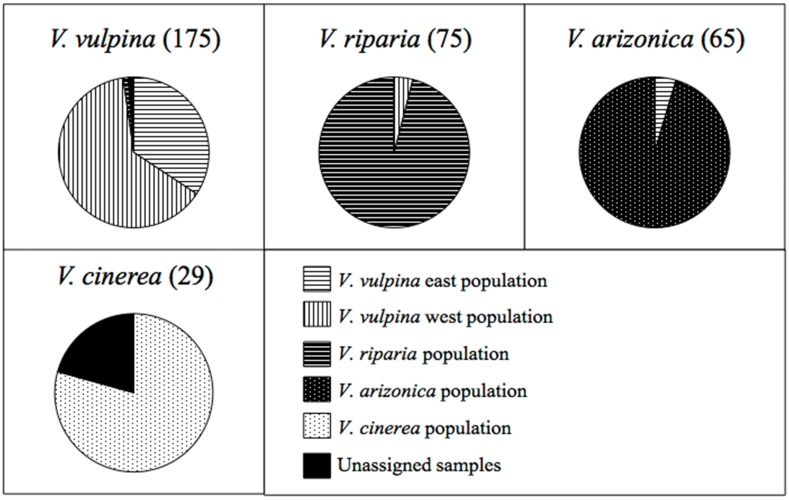
Host based population assignments for the 466 grape phylloxera samples. The number of samples used for each species is in parentheses next to the species name. Coding indicates samples both "in" and "associated" with each population from the STRUCTURE analysis.

A strong host species influence on the population structure of the phylloxera in its native range was observed (Fig 2). Ninety-six percent of samples taken from *V*. *riparia* host plants clustered together in the *V*. *riparia* population. The *V*. *arizonica* populations included 95 percent of the samples collected on *V*. *arizonica*, and 100 percent of the samples collected on *V*. *treleasei* (a glabrous form of *V*. *arizonica*). The *V*. *cinerea* population contained 72 percent of the samples collected on *V*. *cinerea*, and 100 percent of the samples collected on what appeared to be *V*. *cinerea* x *V*. *vulpina* hybrids. Similarly, the *V*. *vulpina* east and *V*. *vulpina* west populations contained 97 percent of the samples collected on *V*. *vulpina*.

Only 16 samples collected across the four main host species appeared to be outliers. Four of the samples collected on *V*. *vulpina* did not cluster with either the *V*. *vulpina* east or west populations. One of the outliers collected on *V*. *vulpina* was collected on the border of the *V*. *vulpina* east and west populations and was identified as an admixture between the two populations by STRUTURE. The remaining three outliers from *V*. *vulpina* all came from one plant collected in Texas and were identified by STRUCTURE as associated with the *V*. *riparia* population or were an admixture of the *V*. *riparia* and *V*. *vulpina* west populations. Three samples collected on *V*. *riparia* clustered with the *V*. *vulpina* west population. One of the samples was from a site in Indiana where members of both the *V*. *riparia* and *V*. *vulpina* west population were identified; while the other two samples were found at a site in Tennessee halfway between Nashville and Memphis. Three of the samples collected on *V*. *arizonica* at one site in Silver City, NM clustered with the *V*. *vulpina* east population. These samples were collected in an urbanized area and these *V*. *arizonica* vines may have been hybrids with other species. Six of the samples collected on *V*. *cinerea* at one site in Florida were all identified by STRUCTURE as being admixtures between the *V*. *cinerea* and *V*. *vulpina* east populations.

### Geographic distribution of populations

**Fig 3:**
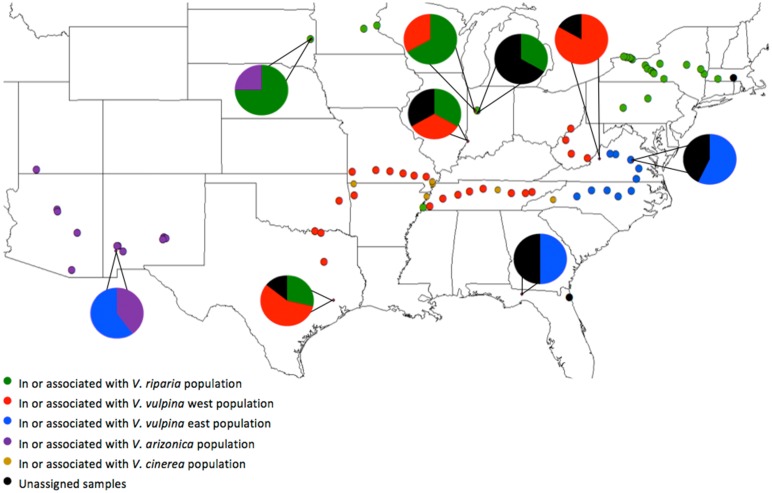
Geographic distribution of the 5 grape phylloxera populations identified in this study. Solid colored circles indicate that all samples collected at that site are in or associated with a particular population according to the STRUCTURE results. Dots with accompanying pie charts denote sites with a mixture of populations in proportions indicated by the pie chart.

A strong correlation between geographic regions and populations was also identified, however, this association was largely the result of the nonrandom distribution of the *Vitis* species sampled in this study (Fig 3). Only samples collected from *V*. *vulpina* were separated by collection site. All *V*. *vulpina* samples collected from Ohio, West Virginia, Virginia, Tennessee, Missouri, Arkansas and Oklahoma on the west side of the Appalachian Mountains were members of the *V*. *vulpina* west population. All samples collected on the east side of the Appalachian Mountains in Virginia and North Carolina were members of the *V*. *vulpina* east population. This observation indicates that the Appalachian Mountains act as a physical barrier between the two populations of phylloxera that have evolved to grow on the same host grape species, *V*. *vulpina*.

The geographic clustering resulted in several additional findings (Fig 3). A hybrid zone was found running through Indiana with samples from both the *V*. *riparia* and *V*. *vulpina* west populations. Samples collected from this hybrid zone area were identified by STRUCTURE as being admixtures of the two populations. Hybrids between the *V*. *vulpina* east and *V*. *cinerea* populations were found at a site in eastern Virginia and two sites in Florida. These results may indicate a level of gene flow between the *V*. *vulpina* east and *V*. *cinerea* populations, which could explain why STRUCTURE initially combined the two populations.

There were four sites where one of the populations appeared to be outside of their geographic region (Fig 3). The first of these is the southernmost collection from *V*. *riparia* along the Mississippi River in Arkansas. This site was a good example of the selection pressure a host can assert on phylloxera. Even though this collection site was far removed from other *V*. *riparia* collections, this phylloxera MLG were still clustered within the *V*. *riparia* population. The second site was northeastern Texas where samples were collected that grouped with the *V*. *riparia* population. At least three grapes species (*V*. *vulpina*, *V*. *aestivalis* and *Muscadinia rotundifolia*) were identified at this collection site. While we did not identify *V*. *riparia* plants in this region, earlier work placed *V*. *riparia* in this part of Texas [22]. The *V*. *vulpina* east population samples found in New Mexico, and the *V*. *arizonica* population samples found in South Dakota, were likely the result of the movement of infested plant material by humans. The *V*. *arizonica* population samples collected in South Dakota were from a vineyard associated with South Dakota State University where phylloxera may have been accidentally introduced. Similarly the samples from the *V*. *vulpina* east population collected in New Mexico were found in downtown Silver City along a city park/river walk where human importation or movement of grapes from the southeastern United States would be possible.

### Population statistics

Values for Nei’s genetic distance among the 5 populations varied from 0.6909 to 1.9360 (Table 2). Interestingly, the *V*. *vulpina* west and *V*. *cinerea* populations had a large genetic distance (1.3781) even though they have a sympatric range. The divergence between the two populations can also be seen in their F_ST_ values (Table 2). The overall range of the pairwise F_ST_ values was between 0.426 and 0.532 indicating that all populations were distinct. The sympatric *V*. *cinerea* and *V*. *vulpina* west populations had a pairwise F_ST_ value of 0.518, the second highest in the study. The mean number of alleles (na) per locus varied between 3.19 and 7.85, while the effective number of alleles (ne) ranged from 1.72 to 2.93 (Table 3). Shannon’s information index (I) ranged between 0.56 and 1.05. For all of these statistics, lower values were observed for the *V*. *cinerea* population that also had a smaller number of unique MLGs associated with it. Observed heterozygosity (Ho) ranged between 0.249 and 0.415, while expected heterozygosity (He) ranged between 0.293 and 0.501. For all five populations He was greater than Ho. The *V*. *arizonica* population had the greatest disparity between He and Ho with a difference of more than 0.2. The remaining populations were more similar with differences in He and Ho values of less than 0.09.

**Table 2:** Nei's genetic distance below the diagonal and pairwise multi-locus F_ST_ values above the diagonal calculated among the five phylloxera populations. Calculations were only made with samples that were placed within each population (a q value above 0.9) based on the STRUCTURE results.

Population	*V*. *riparia*	*V*. *vulpina* West	*V*. *arizonica*	*V*. *vulpina* East	*V*. *cinerea*
*V*. *riparia*	****	0.498	0.468	0.495	0.532
*V*. *vulpina* West	1.8044	****	0.428	0.426	0.518
*V*. *arizonica*	1.2387	0.9699	****	0.436	0.446
*V*. *vulpina* East	1.936	1.0775	1.1187	****	0.44
*V*. *cinerea*	1.313	1.3781	0.6909	0.7982	****

**Table 3:** Mean Number of Alleles (na), mean Number of Effective alleles (ne), Shannon's Information Index (I), mean Observed Heterozygosity (Ho), and mean Expressed Heterozygosity (He) calculated for the five grape phylloxera populations. Calculations were only made with samples that were placed within each population (a q value above 0.9) based on the STRUCTURE results.

Population	No. of Samples	Mean na	Mean ne	Mean I	Ho	He
*V*. *riparia*	121	7	2.93	0.99	0.38	0.464
*V*. *vulpina* west	164	7.85	2.86	1.04	0.377	0.467
*V*. *arizonica*	66	4.69	2.55	0.88	0.222	0.437
*V*. *vulpina* east	75	6.15	2.79	1.05	0.415	0.501
*V*. *cinerea*	32	3.19	1.72	0.56	0.249	0.293

### Reproductive mode and the impact of host plant and climate

The evidence for sexual reproduction was analyzed at multiple levels. Table 4A, 4B and 4C present the reproductive statistics in the 5 populations, 4 host species and samples collected in 16 States, respectively. Table 4D and 4E present the statistics of the 14 State-host combinations and the 15 sites that had at least 8 MLG’s. Hardy Weinberg equilibrium probabilities ranged from 0.013 to 0.613. Only 5 groups (the *V*. *arizonica* population–Table 4A, the samples collected from *V*. *arizonica* and *V*. *vulpina–*Table 4B, and the samples collected in Arkansas and Tennessee–Table 4C) had values below 0.100 and therefore the null hypothesis that these samples are in HW equilibrium could be rejected. Considering the 5 groups with HW probabilities below 0.100, three (the samples collected on *V*. *vulpina* and the samples collected in Arkansas and Tennessee) were identified as having a structured population with multiple sub-populations within the group, a situation that can disrupt HW equilibrium [23]. Removing these three groups from the 5 groups with HW probabilities below 0.100 left the *V*. *arizonica* population and samples collected on *V*. *arizonica* as the only groups where HW equilibrium could be rejected.

**Table 4:** A-E. Reproductive statistics for grape phylloxera groups containing more than 8 Multilocus Genotypes (MLG). Sub-tables are distinguished by: 4A population, 4B host, 4C State, 4D Host within State, 4E Collection Site. Adherence to Hardy-Weinberg (HW) predictions was calculated using G statistics on a per locus basis. Then the mean value for polymorphic loci was calculated for presentation. Multilocus F_IS_ was calculated within each identified group. Clonal diversity (R) was calculated by (G-1)/(N-1), where G is the number of MLGs present in a group and N is the total number of samples.

	# MLGs	HW G^2^	F_IS_	R
4A. Reproductive Statistics by Population				
*V*. *arizonica*	66	0.067	0.494	1
*V*. *cinerea*	32	0.36	0.154	1
*V*. *riparia*	116	0.203	0.18	0.958
*V*. *vulpina* East	65	0.284	0.173	0.865
*V*. *vulpina* West	146	0.295	0.193	0.89
4B. Reproductive Statistics by Host				
*V*. *arizonica*	65	0.03	0.501	1
*V*. *cinerea*	29	0.141	0.238	1
*V*. *riparia*	75	0.128	0.203	0.949
*V*. *vulpina*	175	0.023	0.377	0.879
4C. Reproductive Statistics by State				
AR	19	0.013	0.506	0.947
AZ	39	0.123	0.414	1
FL	12	0.18	0.1	1
IN	14	0.285	0.235	1
MA	10	0.269	0.177	1
MN	9	0.574	-0.016	1
MO	53	0.151	0.371	0.963
NC	30	0.247	0.35	0.853
NM	26	0.145	0.486	1
NY	40	0.386	0.038	0.929
PA	9	0.458	0.103	0.889
SD	16	0.211	0.234	0.938
TN	65	0.087	0.293	0.865
TX	18	0.375	0.19	0.944
VA	45	0.23	0.25	0.88
WV	15	0.504	0.16	0.933
4D. Reproductive Statistics by Host-State				
AR *V*. *cinerea*	11	0.528	0.132	1
AZ *V*. *arizonica*	39	0.123	0.414	1
IN *V*. *riparia*	8	0.263	0.264	1
MA *V*. *riparia*	9	0.519	-0.017	1
MO *V*. *cinerea*	12	0.447	-0.024	1
MO *V*. *vulpina*	26	0.412	0.094	0.962
NC *V*. *vulpina*	24	0.514	0.182	0.821
NM *V*. *arizonica*	26	0.145	0.486	1
NY *V*. *riparia*	40	0.386	0.038	0.929
PA *V*. *riparia*	9	0.458	0.103	0.889
TN *V*. *vulpina*	35	0.374	0.153	0.85
TX *V*. *vulpina*	18	0.375	0.19	0.944
VA *V*. *vulpina*	36	0.356	0.102	0.875
WV *V*. *vulpina*	15	0.504	0.16	0.933
4E. Reproductive Statistics by Site				
AZ-086	14	0.23	0.316	1
AZ-087	11	0.198	0.37	1
AZ-089	11	0.148	0.434	1
MA-008	9	0.519	-0.017	1
MO-074	9	0.613	0.036	1
MO-080	11	0.336	0.01	0.909
NC-051	8	0.533	0.001	0.778
PA-027	8	0.496	0.102	0.875
SD-037	12	0.169	0.305	0.917
TN-059	13	0.52	-0.005	0.923
TN-062	11	0.36	0.104	0.909
TN-066	12	0.186	0.077	0.688
VA-045	10	0.285	-0.108	0.818
VA-049	10	0.522	0.01	0.9
WV-040	10	0.471	0.11	0.9

F_IS_ values were generally positive and ranged between -0.108 and 0.506. Only six groups (*V*. *riparia* from Massachusetts, *V*. *cinerea* from Missouri, Minnesota samples, Massachusetts site-008, Tennessee site-059, and Virginia site-045) had negative F_IS_ values. The highest F_IS_ values were detected in the three individual collection sites in Arizona (Table 4E), samples collected in Arizona and New Mexico (Table 4C), samples collected on *V*. *arizonica* (Table 4B), and the *V*. *arizonica* population (Table 4A), indicating higher population divergence. The population divergence in *V*. *arizonica* population was the greatest as evident from the clonal diversity values. Clonal diversity (R) ranged between 0.688 and 1.000. All groups related to the *V*. *arizonica* and *V*. *cinerea* populations had R-values of 1, indicating that no clonal MLGs were sampled. Additionally, samples from Indiana, Minnesota, northern Massachusetts and southern Florida also had R-values of 1. The lowest R-values (indicating the greatest number of clonal MLGs samples) came from groups associated with North Carolina, Tennessee, and Virginia on *V*. *vulpina* and to a lesser extent from samples collected on *V*. *riparia* in Pennsylvania. Climatic data were also evaluated to determine whether temperature plays any role in increasing or decreasing clonal diversity. No trend was observed as clonal samples were identified in both warm and cold climate sites.

## Discussion

### Genotypic data with more than two alleles

More than two alleles were observed in 134 samples with one or more markers. Occurrence of multiple alleles indicates that either the primer sequences had multiple priming sites due to a lack of sequence specificity, or that genomic regions represented by these primers were duplicated in the grape phylloxera genome, or that the samples were mix of two or more genotypes. Sample contamination could have happened at all stages from phylloxera extractions, DNA isolation and fragment size analysis. Sample contamination had occurred at some point with the original FreR1bulk isolate that was verified by single adult DNA extractions with results suggesting that cross contamination with the AxRR1 line had occurred. When the remaining three SAL lines that showed three alleles (VinR1, FreR2 and 101R2) were retested, genotypic data remained consistent, indicating that these lines have real third allele at the PhyIII_19 marker. The identification of more than two alleles with 20 SSR markers in 134 samples is the first indirect glimpse into the genomic complexity of phylloxera. The grape phylloxera genome is approximately 400 Mb and initiatives are underway to fully sequence the genome [24] to improve our understanding of genome organization, number of genes, gene duplication events, size and amount of repetitive elements.

### Population structure in the native range and influence of host plant and geography

In this study we utilized three different analyses (Model-based Bayesian analysis, PCoA and neighbor-joining tree) to identify the approximate number of genetic clusters within the sample set. The program STRUCTURE [25] has been used to identify populations in the Aphididae family in multiple studies [16, 26, 27, 28]. The results observed here from the three assays corroborated with each other and provided confidence that the 5 identified populations were correct. Two prior studies on phylloxera’s genetic diversity in its native range used RAPD markers and mitochondrial sequencing data and detected three host-based populations of phylloxera [12, 13]. Samples collected from the host plant *V*. *riparia* conform to one population both with RAPD [12] and mitochondrial sequence data [13]. In this study, we also found that all phylloxera samples collected from *V*. *riparia* were placed in the *V*. *riparia* population. This is an important result and shows that phylloxera populations adapted to *V*. *riparia* are stable for at least the 12 years between two studies. The other host-based populations identified earlier were less consistent within those studies [12, 13] and results varied depending on the system (RAPD markers or sequence data) used. In fact, the groups identified with the mitochondrial sequence data were less well organized by either geographic or host-based groupings possibly because of the low sample number. In our study, we identified five clear groupings with four host plants (Fig 1). The host on which the collections were sampled played a major role in the differentiation of the 5 populations, which was particularly clear with the *V*. *riparia* and *V*. *arizonica*- based samples. The concept of host-based selective pressure is common in grape phylloxera both within their natural range [12] and their introduced range [7]. The host-based effect is also common across the closely related Aphididae family and is a defining part of their biology [16, 29, 30]. However, the separation of the *V*. *vulpina*-based samples into east and west populations identified for the first time in this study indicates that the host may not be the only factor distinguishing phylloxera populations.

Separation of host- and geography-based associations within *Vitis* species is not easy. Most *Vitis* species are associated with distinct geographic ranges. For instance, *V*. *riparia* is most commonly found in the north central and northeastern United States and *V*. *arizonica* is restricted to the southwestern United States and northern Mexico. There was a geographic distinction within the samples collected from *V*. *vulpina*. These collection sites were separated by the Appalachian Mountains and were grouped into an eastern and western population. The climatic conditions on either side of the Appalachian range are distinctly different with relatively colder climate on the west side, an additional factor capable of increasing the selective pressure driving divergence and specialization on the same host plant. Geographic correlations were also found in previous studies of grape phylloxera’s native range [12, 13]. In the introduced range this phenomena can also be observed at a small scale within a vineyard [8], and on a regional scale across vineyards [9]. The effect of geography on genetic diversity has also been observed with other members of the Phylloxeridae and closely related Aphididae family [14, 30, 31].

Population statistic comparisons of 5 phylloxera populations also showed distinct patterns. Both F_ST_ and Nei’s genetic distance varied widely among the 5 populations, while other parameters were less variable. When the smaller *V*. *cinerea* population was excluded, the remaining four populations had similar values for the number of alleles, effective alleles and information content of alleles. These results indicate that although the four larger populations are genetically distinct from each other but they have comparable levels of diversity within each. The major differences among the populations were lower observed heterozygosity in comparison to their expected values indicating that inbreeding is playing a role. Specifically, *V*. *arizonica* and *V*. *cinerea*-based populations had significantly lower Ho than He as a result of their more isolated geographic locations and potential for increased inbreeding.

### The introduced range

Determining the level of genetic diversity in the native and introduced range of phylloxera is critical to the development of management strategies. In this study, we sampled extensively across the native range of phylloxera to establish its population structure within the US, and to determine the point of origin of populations introduced to California and other regions around the world. All samples from the introduced range, including Austria, Brazil, Hungary, Uruguay and California (101R1/2 and FreR1/2B), showed association with the *V*. *riparia* population (Fig 1). In an earlier study by Downie et al. [12, 13] phylloxera samples from California, Oregon and Washington grouped with samples from Pennsylvania, New York and other northern States where *V*. *riparia* is common.

In this study, phylloxera from Argentina, Peru and California (AxRR1 and VinR1) appeared to be admixtures based on STRUCTURE and PCoA results (Fig 1). These apparent admixtures could be the result of sexual reproduction between members of the *V*. *riparia* and *V*. *vulpina* east populations. Previous work by Downie [13] using mitochondrial sequence data found a single haplotype for phylloxera from California, Australia, New Zealand and Peru that grouped with samples collected on *V*. *vulpina* along the Atlantic coast. The matching of mitochondrial DNA from the Atlantic coast in Downie’s work [13] and the admixtures of *V*. *riparia* and the *V*. *vulpina* east populations based on SSR markers may indicate the source of these samples. Our results suggest that the root phylloxera samples from Argentina, Peru and the CA SAL lines VinR1 and AxRR1 tested in our study are the result of sexual reproduction between the *V*. *riparia* and *V*. *vulpina* east populations with the female coming from the *V*. *vulpina east* population. The matching of mitochondrial haplotypes was also used to confirm the presence of the same phylloxera clone in California and Peru [13]. Careful comparisons of the results from the two studies suggests that California, Peru and possibly Australia and New Zealand were contaminated with the same phylloxera clone, perhaps by the movement of infested plant material.

Foliar phylloxera has been rare in California, until the discovery of a widespread outbreak of leaf-galling phylloxera that occurred in grape rootstock nursery plantings in Yolo and Solano counties. One hundred and twenty-two of the 170 leaf-galling phylloxera samples tested in a previous study [32] were determined to be the same clone, represented in this study by the WEO4802 sample; the remaining samples were closely related. STRUCTURE analysis determined that the WEO4802 sample was a mixture of the *V*. *riparia* and *V*. *vulpina* west populations, and was similar to phylloxera collected in Indiana—where these two populations overlap. Further work is needed to determine how this phylloxera strain got to California and whether it exists in other parts of the introduced range.

### Reproductive mode in the native range

The occurrence of sexual reproduction was expected in phylloxera’s native range as active sexual morphs have been identified in the southwestern, central and eastern United States [5, 33]. However, no reports are available that provide conclusive evidence of the effect of sexual reproduction on the genetic population structure at any site. On the other hand, studies in the introduced range, Australia [6], Europe [1, 9], and California [10] have found that asexual reproduction is the primary, if not the only means of reproduction. Asexual reproduction leads to negative F_IS_ values, major departures from HW equilibrium and high numbers of clones with low clonal diversity at any one site.

Contrary to what was found in the introduced range, clonal diversity among all groups within the native range was very high, indicating few clonal MLGs even though many of the samples were collected from the same plant, and in many cases from the same leaf. Most of the groupings contained no clonal MLGs at all. The *V*. *vulpina* west and *V*. *vulpina* east populations, especially from sites collected in North Carolina, Virginia and Tennessee had more clonal MLGs (Table 1). This result may be an artifact of the study’s collection period and its overlap with the optimum time for phylloxera development in these States. If collections had been done earlier there may have been greater numbers of clonal types some of which might have been outcompeted and were more rare during our collection trip.

When the clonal samples were excluded from Hardy-Weinberg analysis the results indicated that most of the groups were in HW equilibrium. This conclusion remained true when a high cutoff of 0.1 was used, and resulted in the rejection of the null hypothesis of HW equilibrium in only 5 of the 54 groups. Three of the 5 groups violated HW assumptions because they contained subpopulations. When the larger of these subpopulations were analyzed they were in HW equilibrium. The only population not in HW equilibrium was the *V*. *arizonica* population, which appeared to be highly inbred. The F_IS_ values were highest for the *V*. *arizonica* population and all of its subgroups. These results are likely due to the typically disjointed grape habitat in the southwestern US where grapes are often on mesic mountainsides separated by many kilometers of hot, dry desert in areas known as “sky islands”. These sky islands occur across Arizona, New Mexico, and northern Mexico have been shown to affect population structure in other species [34, 35, 36]; inbreeding is a common consequence of island population genetics [37, 38].

Reproductive statistics indicated common sexual reproduction, which was also supported by the discovery of admixture samples from both the native and introduced range based on STRUCTURE results. The identification of multiple possible sexual offspring between the *V*. *riparia* and *V*. *vulpina* west populations in Indiana, Texas and California, and the identification of multiple possible sexual offspring from the *V*. *cinerea* and *V*. *vulpina* east populations in Virginia and Florida, suggests that these populations freely mate under natural conditions. While many of the other populations were geographically isolated, or were not collected at the boundary between populations, the *V*. *cinerea* and *V*. *vulpina* west populations were collected across a large sympatric range covering parts of three States; yet no admix samples between the two populations were identified. This observation could be due to chance, or we may conclude that these two populations actively avoid mating. Selective mating associated with host specialization has been detected in pea aphids where chemoreceptor genes have been shown to both affect host selection and reproductive isolation [39]. Measures of clonal diversity were used to evaluate the affect of temperature on the mode of reproduction. No definitive effect of temperature was identified within samples collected from either side of Appalachian mountain range; clonal samples were observed in both warm and cold climates.

## Materials and Methods

### Sample collection

Grape phylloxera are abundant insect pests of grapevines. The phylloxera collected for this project were taken from galls on the leaves of grapevines found growing within the right of way of public roads. No permission was required for the collections. Grapevines were a frequent and weedy species at the collection sites. Our phylloxera samples consisted of eggs and adult forms extracted from leaf galls, which numbered between 10 to over 100 per leaf, and were present on thousands of leaves per grapevine. Neither grape phylloxera nor the grapevine species sampled are endangered or protected species.

Foliar galls of grape phylloxera were collected across the northeastern USA, Atlantic coast and central regions of United States during June 2010. Samples were collected about every 80 km over a circuitous 5,000 km route, and all collection sites were on public roads near major roadways. GPS coordinates were recorded for each site and samples were collected within a 100 m radius of that location. Leaf galls were collected from a variety of native *Vitis* species identified based on foliar features. Samples were collected from a maximum of four plants per site and infested leaves were stored in separate 4L sealable plastic bags, and kept on ice for the remainder of the trip. Sample collection was limited across the northeastern region due to late freezing temperatures that killed phylloxera adults and juveniles. Sampling was carried out across both sides of the Appalachian Mountains, and into the coastal plain of Virginia, Tennessee, Arkansas, southern Missouri, Oklahoma and north central Texas. Favorable leaf galling conditions were present from West Virginia to the edge of Oklahoma allowing collections to be taken at 80 km intervals. Collection sites in Texas were more widely spaced as phylloxera leaf galling was less common.

Samples were also collected from central Arizona in 2010 and from eastern Arizona and western New Mexico in 2011. Sampling was more sporadic in these trips, as the region’s dry climate forms natural barriers to the distribution of grape, and thus phylloxera. In addition, several collaborators from New York (Buffalo and the Finger Lakes), Indiana, Minnesota, South Dakota, southern Texas, Utah and western Florida, sent phylloxera samples to include in this study. Samples from Indiana, Minnesota, South Dakota and the western Florida sites were made at local vineyards growing American *Vitis* species and their hybrids and *V*. *vinifera* × American *Vitis* species hybrids. S2 Table provides the GPS coordinates of all samples collected in this study. The map in Fig 3 was created using ArcGIS^®^ and ArcMap^™^ software by Esri (http://www.esri.com).

To get a representation of phylloxera from other countries, samples were obtained from Europe (Hungary and Austria) and South America (Argentina, Brazil, Peru, and Uruguay). With the exception of Argentina, samples from other South American countries arrived as dried DNA pellets extracted from foliar phylloxera taken from leaf galls. These samples were re-suspended in 60μl of pure water prior to being stored at -20°C. Samples from Europe and Argentina arrived as multiple adults collected from roots and suspended in alcohol.

### Host adapted strains as reference samples

Six single adult lineage (SAL) lines were developed from root feeding behavior experiments on various rootstock hosts in California. Each line was started with single adult phylloxera growing on an excised root in a petri dish following procedures modified from De Benedictis and Granett [40]. The eggs were transferred to fresh root segments every week and allowed to mature into new adults, which then had their eggs transferred to plates of fresh excised roots. This allowed for the production of hundreds of genetically identical individuals originating from a single lineage. These lines served two purposes: to represent different California phylloxera strains, and to provide a source of genetically pure phylloxera DNA to be used as references for genotyping in different studies. The six SAL lines were collected from different hosts and regions in California. The VinR1 line was gathered from the roots of *V*. *vinifera* cv. Chardonnay at the University of California, Davis vineyard. AxR-R1 was collected from the roots of AxR#1 rootstock in Mendocino County. 101-R1 and 101-R2 were collected from the roots of the rootstock 101-14Mgt from different vineyards in Sonoma County. Fre-R1 and Fre-R2 were collected from the rootstock Freedom in separate Napa County vineyards. The WEO4802 sample was collected from a foliar gall on St. George rootstock at the National Clonal Germplasm Repository, Winters CA.

### DNA isolation and genotyping

Individual galls on each leaf were opened with sterile equipment to examine their contents with 10X magnification. Ideally, galls containing one adult with 10 to 100 eggs were collected for DNA extraction. If galls with single adults were unavailable, galls containing multiple adults or galls containing only crawlers were used. The contents from selected galls were placed in a 1.5mL centrifuge tube and stored in a -20°C freezer until DNA extraction. Whenever possible 3, and up to 10, samples were isolated from each sample bag. Bags were kept sealed at all times to avoid cross contamination and left over plant material was autoclaved to prevent dispersal of phylloxera. The SAL phylloxera samples were composed of 50 or more adults from multiple generations. DNA was extracted from all samples using the protocol described in Lin and Walker [41]. After DNA extraction samples were stored at -20°C for further use.

A total of 540 samples were genotyped with 32 fluorescently labeled (6-FAM, HEX, VIC, or NED) simple sequence repeat (SSR) primers published in previous studies [7, 10, 42]. Six California SAL lines (AxR-R1, Vin-R1, Fre-R1, Fre-R2, 101-R2 and WEO4802) were used as reference samples in each plate for consistent genotyping. PCR amplifications were performed in 10μl reactions consisting of 10 ng template DNA, 5 pmoles of each primer, 2.5 mM of each NTP, 1μl 10x gold PCR buffer (Perkin Elmer, Waltham, Massachusetts), 0.05 unit AmpliTaq Gold DNA polymerase (Perkin Elmer) and 2 mM MgCl_2_ solution. All SSR primers were amplified at a 56°C annealing temperature, keeping all other conditions of the protocol constant: 10 minutes at 95°C; 35 cycles of 45 s at 92°C, 45 s at 56°C, 1 minute at 72°C; with a final extension of 10 minutes at 72°C.

PCR products of up to four primers were mixed taking into account label color and fragment size. One μl of mixed products was added to 11μl of HD-formamide and 0.2μl of the internal size standard GeneScan-500 Liz (Life Technologies, Carlsbad, California, USA). The mixture was denatured at 92°C for 2 minutes prior to being run on an ABI 3500 Genetic Analyzer (Applied Biosystems, Foster City, CA) using a 50 cm capillary filled with POP-7 polymer (Applied Biosystems, Foster City, CA). Allele sizes were determined using GeneMapper 4.1 software (Applied Biosystems Co., Ltd., USA).

### Preparation of dataset for analysis

Six markers were discarded due to missing data with more than 20% of samples, and/or inconsistencies in genotyping due to one base pair allelic variation and weak amplifications in different PCR groups. Many samples showed a third allele with one or more markers. In that case, the data were considered missing for that genotype at that marker. In the next step, all samples that had missing data for more than three markers were removed. The FreR1 line was found to be mix of two separate multi-locus genotype (MLG), and was replaced with FreR1A and FreR1B to identify two separate MLGs. The microsatellite tool kit software [43] was used to identify matching/clonal types in the remaining 502 samples.

To investigate whether matching/clonal samples were the result of asexual reproduction (a true clone) or the result of independent sexual events the program MLGsim [44] was used to calculate the probability of sex (P_sex_). Because the program is unable to accommodate missing data, two runs were performed. The initial run consisted of the 215 samples with no missing data at 26 loci. The second run consisted of the remaining 286 samples and 22 loci; four markers (PhyII_10, PhyII_23, PhyIII_19, PhyIII_65) were removed due to missing data. The program was run using both HWE and FIS models at 1,000 simulations each. Analysis of this set identified seven possible clonal MLGs.

### Population structure analysis

The software STRUCTURE V2.3.1 [28] was used to infer the number of genetic clusters in the set of 466 unique MLGs. The membership grouping of each sample was run for a range of genetic clusters (K) with values from 1 to 10 using the admixture model, and the runs were replicated 10 times for each K. Each run used a burn-in cycle of 250,000 steps followed by 500,000 Monte Carlo Markov Chain replicates. The number of clusters was calculated using the delta K method described in Evanno et al. [45], and by assessing the plateau point of the Ln P (D) values.

Samples were considered “in” a cluster when the q value generated from STRUCTURE was 0.90 or above. Samples were considered to be “associated with” a genetic cluster if their q value was between 0.70 and 0.89. To evaluate the STRUCTURE output results, sample sets were also analyzed with the DARwin software [46]. The dissimilarity matrix was used to construct a neighbor-joining tree and to produce a Principal Coordinate Analysis (PCoA) [47]. The three outcomes (STRUCTURE, a neighbor-joining tree and PCoA) were then used to compare the results.

A total of 175 samples showed membership in two populations based on the assigned q-values from the first STRUCTURE run. A second STRUCTURE run was carried out to better assess the clusters within these two populations in the absence of outliers. The criteria described above were used except that the K value ranged from 1 to 6. The number of clusters was determined by both delta K and the plateau point of the Ln P(D) and groupings were also confirmed with a neighbor-joining tree and PCoA. The results of the two STRUCTURE runs were used to color code the samples to reflect their population assignments. The neighbor-joining tree and PCoA analyses were used to verify the refined genetic clusters in the entire set of 466 MLGs and to display the results.

### Comparison of populations

A comparison of the geographic range and host-based association was conducted using species assignment given to the host plants when collected. To test for differences among the populations. Nei’s genetic distance [48] was calculated using POPGENE version 1.31 [49]. Pairwise multilocus F_ST_ values [50] between all population pairs was also calculated using FSTAT V2.9.3.2 [51] to evaluate the genetic diversity among the populations. Observed heterozygosity (Ho), expected heterozygosity (He), number of alleles (Na), number of effective alleles (Ne), and Shannon’s information index (I) were all calculated for a single locus using POPGENE version 1.31. The mean response across all loci was calculated for comparison.

### Reproductive mode

Phylloxera’s reproductive mode was investigated and compared within groups delineated by population, collection site, State, host, and hosts within States. Any group with less than 8 MLGs was excluded from the analysis. Clonal diversity (R) of each group was calculated as (G-1)/(N-1) where G was the number of MLGs present inside of a specific group and N was the total number of samples in that group [52]. Clonal diversity could range from 1, indicating that there were no clonal samples in a test group, to 0, indicating that all samples belonged to the same clonal MLG (assuming that asexual reproduction was responsible for this). Next, multilocus F_IS_ [50] was tested for each group using the program FSTAT V2.9.3.2. Lastly, Hardy-Weinberg Equilibrium (HWE) was tested for goodness of fit with G-statistics for each locus using POPGENE version 1.31. Mean response across all polymorphic loci was calculated for comparison. A cut off point of 0.1 was used for the acceptance of the null hypothesis of HW equilibrium. Clonal samples from within a collection site were again removed for the calculation of HW equilibrium and F_IS_.

### Impact of climate on reproduction

Weather stations closest to each collection point were identified and records were downloaded from the National Climatic Data Center (NCDC) a unit of the National Oceanic and Atmospheric Administration (NOAA). Weather reports containing the average minimum and maximum temperature per month averaged between 1981 and 2010 for the identified weather station were obtained from an online database (http://www.ncdc.noaa.gov/data-access/land-based-station-data/land-based-datasets/climate-normals/1981-2010-normals-data). Clonal diversity was then compared to the lowest minimum and highest maximum temperatures for each collection site.

## Supporting Information

S1 Table List of samples that had showed more than two alleles with different SSR markers. (XLSX)

S2 Table SSR allelic data of the 466 unique MLGs identified in this study. ND reflects no data.
(XLSX)

S1 Fig Principal Coordinate Analysis (PCoA) of the 466 unique MLGs with color-coding to depict a K = 5 STRUCTURE outcome. The X-axis accounts for 21% of the variation, while the Y-axis accounts for 8.27%. Each population is circled and labeled with a corresponding color. Samples in triangles were not considered part of the *V*. *vulpina* west population by STRUCTURE, but were grouped with the population in the neighbor-joining tree and PCoA. Samples in squares were considered to be part of the *V*. *riparia* population by STRUCTURE, but were not grouped with the population in the neighbor-joining tree and PCoA. All admixed samples that were not placed in any one population are coded by black color.
(TIF)

## References

[1] Vorwerk S, Forneck A. Reproductive mode of grape phylloxera (*Daktulosphaira vitifoliae*, Homoptera: Phylloxeridae) in Europe: molecular evidence for predominantly asexual populations and a lack of gene flow between them. Genome. 2006; 49: 678–687. 10.1139/g06-028 16936847

[2] Forneck A, Huber L. (A)sexual reproduction—a review of life cycle of grape phylloxera, *Daktulosphaira vitifoliae*. Entomologia Experimentalis et Applicata. 2008; 131: 1–10.

[3] Powell KS. Grape phylloxera: an overview In: editors. Johnson SN, Murray PJ, Root Feeders: An Ecosystem Perspective; 2008 pp. 96–114.

[4] Granett J, Walker MA, Kocsis L, Omer AD. Biology and management of grape phylloxera. Annual Review of Entomology. 2001; 46: 387–412. 10.1146/annurev.ento.46.1.387 11112174

[5] Downie DA, Granett J, Fisher JR. Distribution and abundance of leaf galling and foliar sexual morphs of grape phylloxera (Hemiptera: Phylloxeridae) and *Vitis* species in Central and Eastern United States. Environmental Entomology. 2000; 29: 979–986.

[6] Corrie AM, Crozier RH, van Heeswijck R, Hoffmann AA. Clonal reproduction and population genetic structure of grape phylloxera, *Daktulosphaira vitifoliae*, in Australia. Heredity. 2002; 88: 203–211. 10.1038/sj.hdy.6800028 11920122

[7] Corrie AM, van Heeswijck R, Hoffmann AA. Evidence for host-associated clones of grape phylloxera *Daktulosphaira vitifoliae* (Hemiptera: Phylloxeridae) in Australia. Bulletin of Entomological Research. 2003; 93: 193–201. 10.1079/BER2003232 12762861

[8] Corrie AM, Hoffmann AA. Fine-scale genetic structure of grape phylloxera from the roots and leaves of *Vitis*. Heredity. 2004; 92: 118–127. 10.1038/sj.hdy.6800393 14679391

[9] Forneck A, Walker MA, Blaich R. Genetic structure of an introduced pest, grape phylloxera (*Daktulosphaira vitifoliae* Fitch), in Europe. Genome. 2000; 43: 669–678. 10984180

[10] Lin H, Walker MA, Hu R, Granett J. New simple sequence repeat loci for the study of grape phylloxera (*Daktulosphaira vitifoliae*) genetics and host adaptation. American Journal of Enology and Viticulture. 2006; 57: 33–40.

[11] Granett J, Timper P. Demography of grape phylloxera, *Daktulosphaira vitifoliae* (Homoptera: Phylloxeridae), at different temperatures. Journal of Economic Entomology. 1987; 80: 327–329.

[12] Downie DA, Fisher JR, Granett J. Grapes, galls and geography: the distribution of nuclear and mitochondrial DNA variation across host-plant species and regions in a specialist herbivore. Evolution. 2001; 55: 1345–1362. 11525459

[13] Downie DA. Locating the source of an invasive pest, grape phylloxera, using a mitochondrial DNA gene genealogy. Molecular Ecology. 2002; 11: 2013–2026. 12296945 10.1046/j.1365-294x.2002.01584.x

[14] Miller NJ, Birley AJ, Overall ADJ, Tatchell GM. Population genetic structure of the lettuce root aphid, *Pemphigus bursarius* (L.), in relation to geographic distance, gene flow and host plant usage. Heredity. 2003; 91: 217–223. 10.1038/sj.hdy.6800331 12939621

[15] Dickey AM, Medina RF. Host-associated genetic differentiation in pecan leaf phylloxera. Entomologia Experimentalis et Applicata. 2012; 143: 127–137.

[16] Jaquiery J, Stoeckel S, Nouhaud P, Mieuzet P, Maheo F, Legeai F, et al Genome scans reveal candidate regions involved in the adaptation to host plant in the pea aphid complex. Molecular Ecology. 2012; 21: 5251–5264. 10.1111/mec.12048 23017212

[17] Lin H, Downie DA, Walker MA, Granett J, English-Loeb G. Genetic structure in native populations of grape phylloxera (Homoptera: Phylloxeridae). Annals of the Entomological Society of America. 1999; 92: 376–381.

[18] Lund KT. Phylloxera Biodiversity. Ph.D. Dissertation, University of California, Davis. 2013

[19] Lider L. Phylloxera-resistant grape rootstocks for the coastal valleys of California. Hilgardia. 1958; 27: 287–317.

[20] Granett J, Timper P, Lider LA. Grape phylloxera (*Daktulosphaira vitifoliae*) (Homoptera: Phylloxeridae) biotypes in California. Journal of Economic Entomology. 1985; 78: 1463–1467.

[21] Herbert KS, Umina PA, Mitrovski PJ, Powell KS, Viduka K, Hoffmann AA. Clone lineages of grape phylloxera differ in their performance on *Vitis vinifera*. Bulletin of Entomological Research. 2010; 100: 671–678. 10.1017/S0007485310000027 20482931

[22] Munson TV. Foundation of American Grape Culture. TV Munson and Son, Denison Texas; 1909

[23] Sha Q, Zhang S. A test of Hardy-Weinberg equilibrium in structured populations. Genetic Epidemiology. 2011; 35: 671–678. 10.1002/gepi.20617 21818775

[24] Delmotte F, Forneck A, Powell K, Rispe C, Tagu D. Proposal to sequence the genome of the grape Phylloxera (*Daktulosphaira vitifoliae* Fitch). 2014, http://bipaa.genouest.org/is/wp-content/uploads/2015/10/White-Paper-Phylloxera_25may2011-1.pdf

[25] Pritchard JK, Stephens M, Donnelly P. Inference of population structure using multilocus genotype data. Genetics. 2000; 155: 945–959. 10835412 10.1093/genetics/155.2.945 PMC1461096

[26] Cao J, Li J, Niu J, Liu X, Zhang Q. Population structure of *Aphis spiraecola* (Hemiptera: Aphididae) on pear trees in China identified using microsatellites. Journal of Economic Entomology. 2011; 105: 583–591. 10.1603/ec11368 22606830

[27] Ferrari J, West JA, Via S, Godfray HCJ. Population genetic structure and secondary symbionts in host-associated populations of the pea aphid complex. Evolution. 2012; 66: 375–390. 10.1111/j.1558-5646.2011.01436.x 22276535

[28] Zhang B, Edwards OR, Kang L, Fuller SJ. Russian wheat aphids (*Diuraphis noxia*) in China: native range expansion or recent introduction? Molecular Ecology. 2012; 21: 2130–2144. 10.1111/j.1365-294X.2012.05517.x 22417053

[29] Carletto J, Lombaert E, Chavigny P, Brevault T, Lapchin L, Vanlerberghe-Masutti F. Ecological specialization of the aphid *Aphis gossypii* Glover on cultivated host plants. Molecular Ecology. 2009; 18: 2198–2212. 10.1111/j.1365-294X.2009.04190.x 19635073

[30] Peccoud J, Figueroa CC, Silva AX, Ramirez CC, Mieuzet L, Bonhomme J, et al Host range expansion of an introduced insect pest through multiple colonizations of specialized clones. Molecular Ecology. 2008: 17: 4608–4618. 10.1111/j.1365-294X.2008.03949.x 19140984

[31] Guillemaud T, Blin A, Simon S, Morel K, Franck P. Weak spatial and temporal population genetic structure in the rosy apple aphid, *Dysaphis plantaginea*, in French apple orchards. PLoS ONE. 2011; 6: e21263 10.1371/journal.pone.0021263 21701679 PMC3119056

[32] Riaz S, Lund KT, Walker MA. Population diversity of grape phylloxera in northern California and evidence for sexual reproduction. American Journal of Enology and Viticulture. 2016;

[33] Downie DA, Granett J. A life cycle variation in grape phylloxera *Daktulosphaira vitifoliae* (Fitch). Southwestern Entomologist. 1998; 23: 11–16.

[34] McCormack JE, Bowen BS, Smith TS. Integrating paleoecology and genetics of bird populations in two sky island archipelagos. BMC Biology. 2008; 6:28 10.1186/1741-7007-6-28 18588695 PMC2474579

[35] Tennessen JA, Zamudio KR. Genetic differentiation among mountain island populations of the striped plateau lizard, *Sceloporus virgatus* (Squamata: Phrynosomatidae). Copeia. 2008; 3: 558–564.

[36] Mitchell SG, Ober KA. Evolution of *Scaphinotus petersi* (Coleoptera: Carabidae) and the role of climate and geography in the Madrean sky islands of southeastern Arizona, USA. Quaternary Research. 2013; 79: 274–283.

[37] Clarke LJ, Whalen MA, Mackay DA. Cutting grass on desert islands: genetic structure of disjunct coastal and central Australian populations of *Gahnia trifida* (Cyperaceae). Journal of Biogeography. 2013; 40: 1071–1081.

[38] Woolaver LG, Nichols RK, Morton ES, Stutchbury BJM. Population genetics and relatedness in a critically endangered island raptor, Ridgway’s Hawk *Buteo ridgwayi*. Conservation Genetics. 2013; 14: 559–571.

[39] Smadja CM, Canbäck B, Vitalis R, Gautier M, Ferrari J, Zhou JJ, et al Large-scale candidate gene scan reveals the role of chemoreceptor genes in host plant specialization and speciation in the pea aphid. Evolution. 2012; 66: 2723–2738. 10.1111/j.1558-5646.2012.01612.x 22946799

[40] De Benedictis JA, Granett J. Variability of responses of grape phylloxera (Homoptera: Phylloxeridae) to bioassays that discriminate between California biotypes. Journal of Economic Entomology. 1992; 85: 1527–1534.

[41] Lin H, Walker MA. Extraction of DNA from eggs of grape phylloxera (*Daktulosphaira vitifoliae* Fitch) for use in RAPD testing. Vitis. 1996; 35: 87–89.

[42] Riaz S, Lin H, Lund KT, Walker MA. Development and characterization of 89 microsatellite markers for grape phylloxera *Daktulosphaira vitifoliae*. Vitis. 2014; 53: 95–101.

[43] Park SDE. Trypanotolerance in West African cattle and the population genetic effects of selection. Ph.D. thesis. Trinity College Dublin, Dublin, Ireland. 2001.

[44] Stenberg P, Lundmark M, Saura A. MLGsim: a program for detecting clones using a simulation approach. Molecular Ecology Notes. 2003; 3: 329–331.

[45] Evanno G, Regnaut S, Goudet J. Detecting the number of clusters of individuals using the software STRUCTURE: a simulation study. Molecular Ecology. 2005; 14: 2611–2620. 10.1111/j.1365-294X.2005.02553.x 15969739

[46] Perrier X, Jacquemoud-Collet JP. DARwin software. 2006. http://darwin.cirad.fr/

[47] Perrier X, Flori A, Bonnot F. Data analysis methods In: editors. Hamon P, Seguin M, Perrier X, Glaszmann JC, Genetic Diversity of Cultivated Tropical Plants; 2003 pp. 43–76.

[48] Nei M. Estimation of average heterozygosity and genetic distance from a small number of individuals. Genetics. 1978; 89: 583–590. 17248844 10.1093/genetics/89.3.583 PMC1213855

[49] Yeh FC, Boyle TJB. Population genetic analysis of co-dominant and dominant markers and quantitative traits. Belgian Journal of Botany. 1997; 129: 157.

[50] Weir BS, Cockerham CC. Estimating F-statistics for the analysis of population structure. Evolution. 1984; 38: 1358–1370. 28563791 10.1111/j.1558-5646.1984.tb05657.x

[51] Goudet J. Fstat version 1.2: a computer program to calculate F-statistics. Journal of Heredity. 1995; 86: 485–486.

[52] Sandrock C, Razmjou J, Vorburger C. Climate effects on life cycle variation and population genetic architecture of the black bean aphid, *Aphis fabae*. Molecular Ecology. 2011; 20: 4165–4181. 10.1111/j.1365-294X.2011.05242.x 21883588

