# Supplementary figures and images for: Population Structure, Diversity and Reproductive Mode of the Grape Phylloxera (Daktulosphaira vitifoliae) across Its Native Range
Source: PLoS One. 2017 Jan 26;12(1):e0170678. doi: 10.1371/journal.pone.0170678 (PMC5268464; doi:10.1371/journal.pone.0170678)

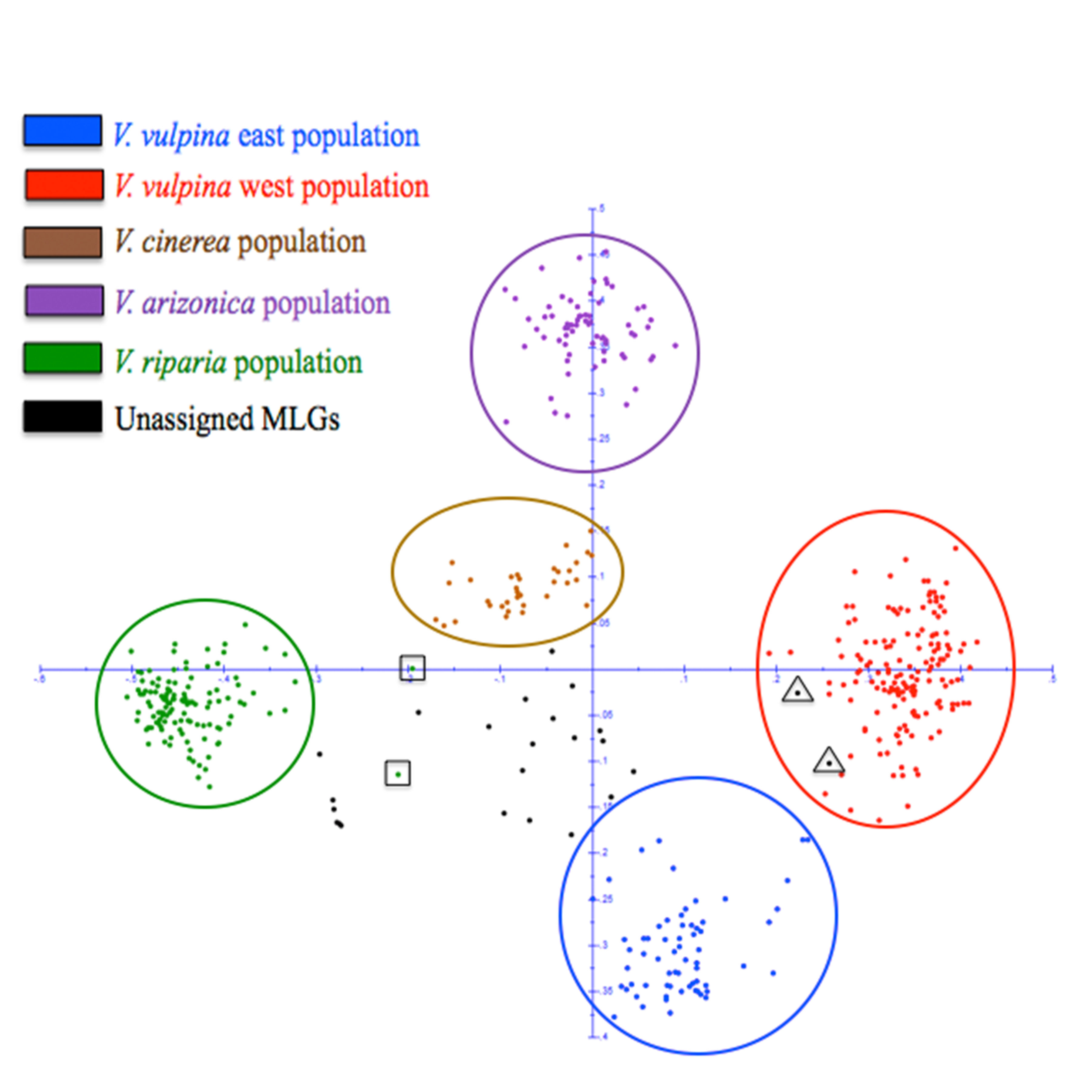

Supplement: S1 Fig — The X-axis accounts for 21% of the variation, while the Y-axis accounts for 8.27%. Each population is circled and labeled with a corresponding color. Samples in triangles were not considered part of the V. vulpina west population by STRUCTURE, but were grouped with the population in the neighbor-joining tree and PCoA. Samples in squares were considered to be part of the V. riparia population by STRUCTURE, but were not grouped with the population in the neighbor-joining tree and PCoA. All admixed samples that were not placed in any one population are coded by black color. (TIF) [file pone.0170678.s003.tif]
